# Postoperative Tapia syndrome after lumbar posterior lumbar interbody fusion: a case report

**DOI:** 10.3389/fmed.2026.1802688

**Published:** 2026-03-25

**Authors:** Jinshuai Zhai, Bo Gao, Teng Huang, Xiao Zheng, Long Zhang, Wenyi Li

**Affiliations:** The Department of Orthopedics, Hebei General Hospital, Shijiazhuang, Hebei, China

**Keywords:** case report, herniated disc, lumbar spine, PLIF, Tapia syndrome

## Abstract

Tapia syndrome is a rare complication characterized by concomitant injury to the hypoglossal and vagus nerves, most commonly associated with airway procedures during general anesthesia. Its incidence is extremely low. Literature review reveals that most reported cases involve Tapia syndrome following posterior cervical spine surgery, with no documented cases of Tapia syndrome occurring after open posterior lumbar spine surgery. We report a case of a patient with intradural lumbar disc herniation who underwent routine lumbar PLIF (posterior lumbar interbody fusion) under general anesthesia. The surgery was uneventful with satisfactory clinical outcomes. However, postoperative hoarseness and tongue deviation were observed. Fiberoptic laryngoscopy revealed fixed abduction of the right vocal cord with normal mobility on the left side, suggesting right vocal cord paralysis and leading to a diagnosis of Tapia syndrome. Active management included neurotrophic agents, glucocorticoids, anti-edema medications, and a rehabilitation plan featuring articulation therapy. At the 2-year postoperative follow-up, hoarseness improved by 90%. Although Tapia syndrome following lumbar surgery is extremely rare, enhancing awareness and understanding of this condition is crucial. Particular attention should be given to patients presenting with hoarseness and deviated tongue protrusion after general anesthesia, ensuring timely detection, prompt diagnosis, and early intervention. This rare case (post-lumbar <5 reported) highlights prone positioning risks; vigilant monitoring and early SLP referral essential; conservative management effective in transient cases. This approach helps prevent misdiagnosis and missed diagnosis, thereby minimizing the adverse impact of complications on patients.

## Introduction

Tapia syndrome was first described in 1904 by Spanish otolaryngologist Antonio García Tapia. It refers to complications arising from damage to the laryngeal recurrent branch of the vagus nerve and the hypoglossal nerve. Clinical manifestations include vocal cord paralysis, hoarseness, dysphagia, dysarthria, deviated tongue protrusion, and tongue atrophy (1). This syndrome may occur in conditions such as atlantoaxial fractures/dislocations or tumors, due to the unique anatomical location of the involved nerves. These nerves traverse the lateral wall of the hypopharynx (base of the tongue) and the epiglottis (pyramidal fossa) region. Consequently, prolonged pharyngeal pressure (e.g., from tumor compression) may trigger Tapia syndrome (2). The occurrence of Tapia syndrome following endotracheal intubation under general anesthesia is extremely rare in clinical practice. A literature review reveals only sporadic case reports of Tapia syndrome during spinal surgery, all involving posterior cervical procedures. To date, no reports exist of this syndrome occurring after open posterior lumbar surgery. This paper reports a case of Tapia syndrome occurring after posterior lumbar interbody fusion (PLIF) under general anesthesia in a patient with an intradural lumbar disc herniation.

## Case presentation

**Figure 1:**
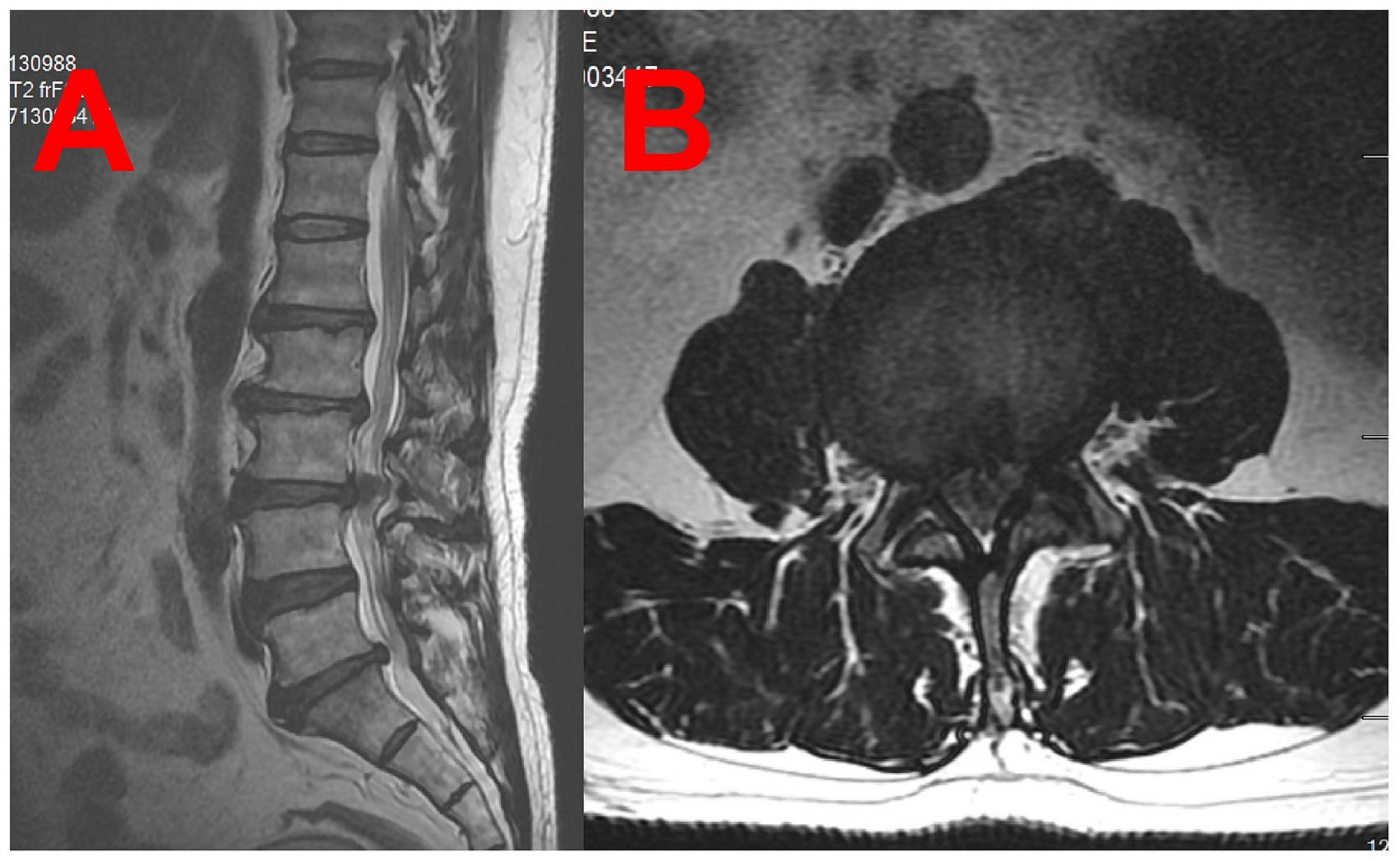
**(A,B)** Lumbar MRI revealed a herniated disc between L3 and L4, compressing the dural sac.

**Figure 2:**
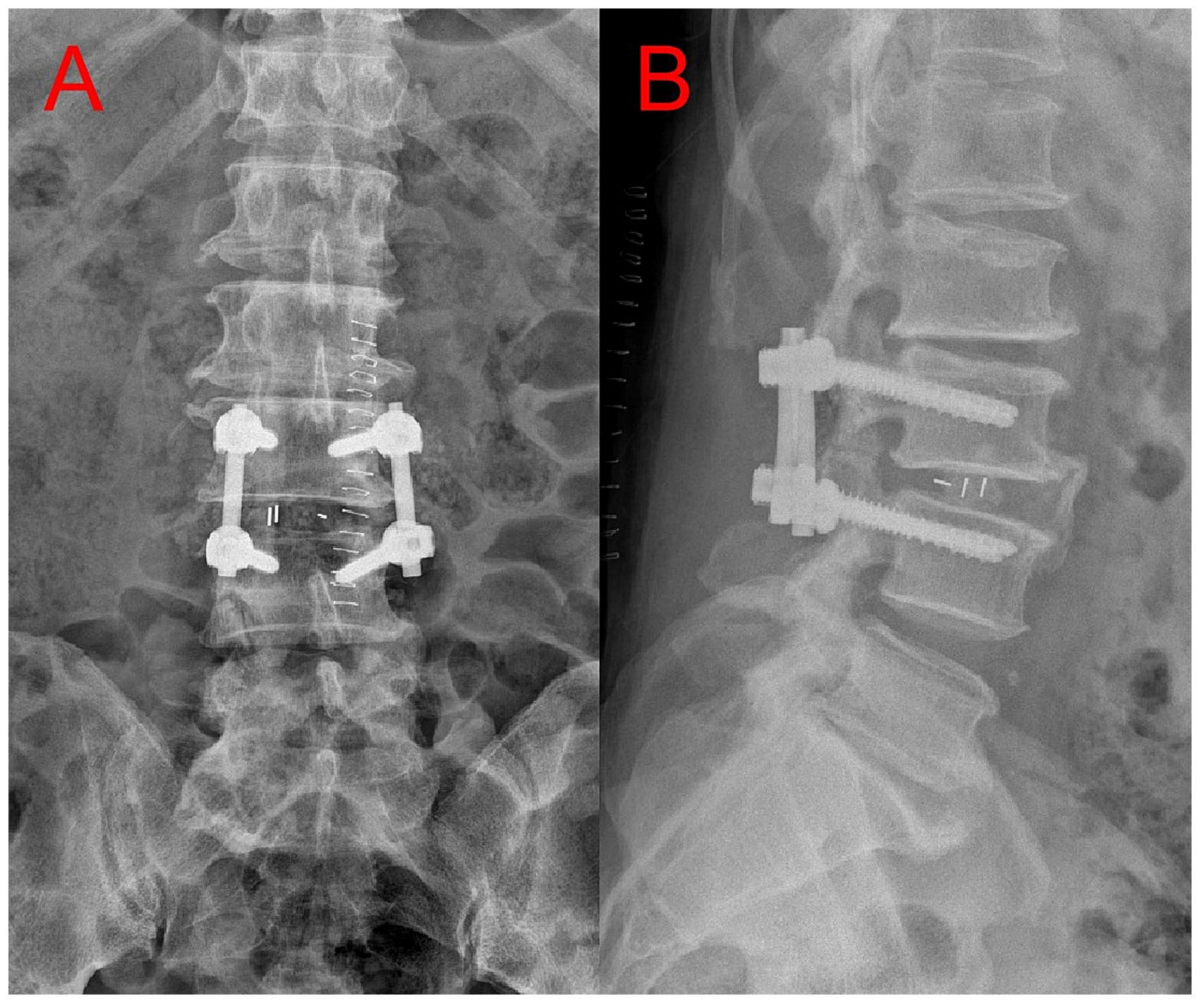
**(A,B)** Lumbar spine X-ray (1 week postoperatively) indicate adequate decompression with the internal fixation devices positioned at optimal angles.

**Figure 3:**
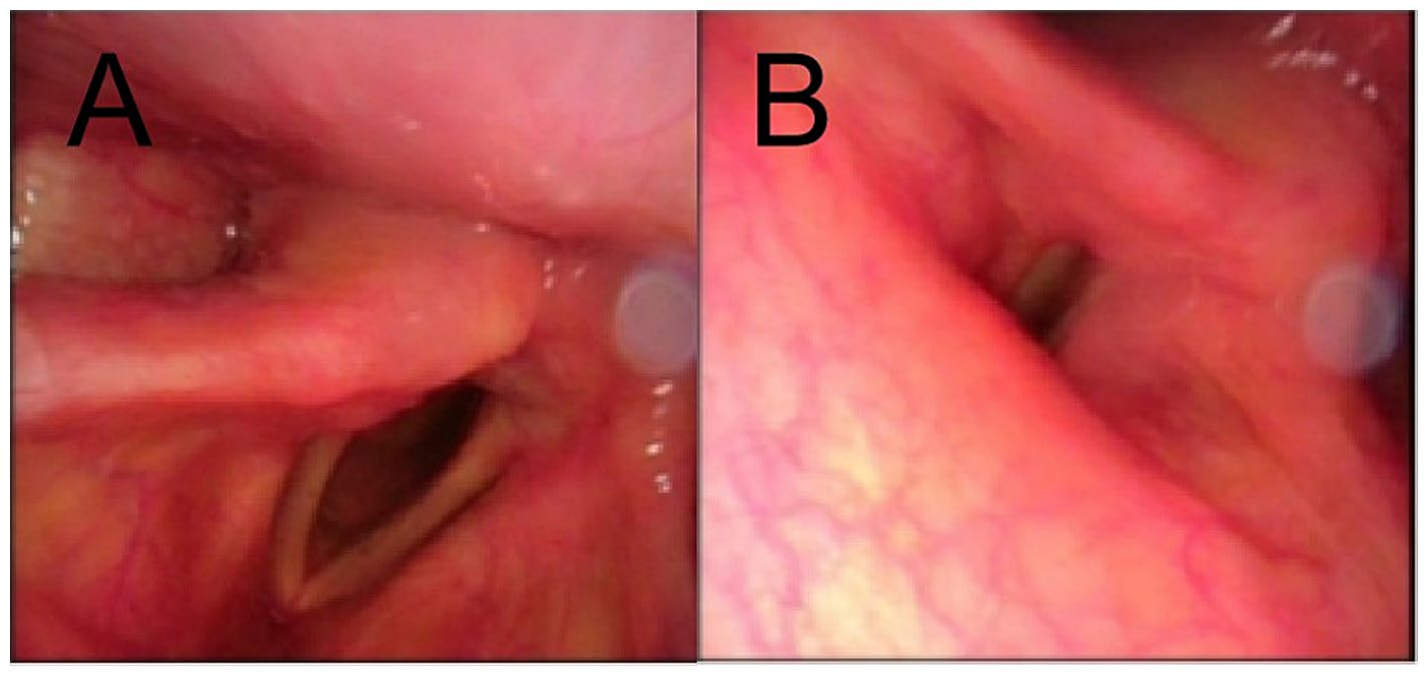
**(A,B)** Electronic laryngoscopy suggested fixation of the right vocal fold abduction, good left activity, and poor closure of the vocal folds.

A 52-year-old male patient with a BMI of 29.7 was diagnosed with an intradural lumbar disc herniation (Figures 1A,B). The patient is an Asian male farmer. He had a history of diabetes mellitus with well-controlled blood glucose levels. He had no significant surgical history. He has no family history of any diseases. No h/o of long term medication and no known allergies till date. Does not consume alcohol, non smoker. Preoperative examinations revealed no abnormalities and no contraindications for surgery. Preoperative routine assessment was conducted, including completion of anesthesia risk evaluation. ASA physical status: Grade II. Airway assessment (Mallampati classification): Grade II. The patient underwent lumbar PLIF surgery (Figures 2A,B) under general anesthesia in the prone position. Anesthetic induction was performed prior to endotracheal intubation, with sequential intravenous administration of the following agents: midazolam injection 2 mg, etomidate emulsion injection 22 mg, sufentanil citrate injection 30 μg, and vecuronium bromide injection 20 mg. Once conditions for intubation were met (mandibular relaxation, loss of consciousness, cessation of breathing), the anesthesiologist initiated the intubation procedure: Position the patient appropriately, open the mouth, spray the pharynx with 4 mL of 2% lidocaine injection, then insert a disposable laryngoscope (Cormack–Lehane airway assessment Grade: I). With clear visualization of the epiglottis and glottis, advance a 7.5 mm endotracheal tube through the glottis into the trachea to a depth of 22 cm. After confirming tracheal placement, secure the tube properly. The entire intubation process proceeded smoothly, achieving success in a single attempt. The surgery lasted 165 min with approximately 400 mL of intraoperative blood loss. Upon regaining consciousness postoperatively, the patient returned to the ward without discomfort. On postoperative day 1, during morning rounds, the patient reported hoarseness hoarseness and dysphagia consistent with vagus nerve involvement. By afternoon, the patient developed lateral deviation of the tongue, consistent with right hypoglossal nerve palsy. An urgent neurology consultation was requested. Physical examination: Alert, with dysarthria; bilateral pupils equal in size and round, diameter 3.0 mm, with intact pupillary light reflexes; symmetrical facial features, tongue protrusion deviated. Muscle strength in all limbs unchanged from previous assessment, muscle tone not increased. And cranial CT/MRI was performed, revealing no evidence of new cerebral infarction. An anesthesiology consultation concluded that tracheal intubation had proceeded uneventfully, with no immediate intervention required. On postoperative day 2, with no improvement in symptoms, an ENT consultation was requested. CT of the arytenoid-styloid joint showed no dislocation but irregular right vocal cord morphology. Under local anesthesia, electronic fiber laryngoscopy revealed that the mucosa of both arytenoid cartilages and vocal cords was smooth. The right vocal cord was in a fixed abducted position, while the left vocal cord had good mobility. Both vocal cords had poor closure, indicating right vocal cord paralysis (Figures 3A,B). The patient experience transient dysphagia postoperatively, lasting for 2–3 days. After receiving prompt symptomatic treatment, the dysphagia resolved rapidly. Following a multidisciplinary consultation and considering the patient’s condition, Tapia syndrome was suspected. Active treatment included neurotrophic agents, glucocorticoids, and anti-edema medications. A rehabilitation plan was established, incorporating articulation disorder training. At discharge on postoperative day 13, hoarseness and lateral deviation of the tongue showed improvement. Post-discharge treatment continued. Post-discharge treatment includes: ① Continued neurotrophic agent therapy: Vitamin B1, orally administered at 10 mg per dose, three times daily, and maintained until 3 months postoperatively. Cobalamin tablets, orally administered at 0.5 mg per dose, three times daily, and continued until 9 months postoperatively. ② After discharge, the patient will continue to receive functional rehabilitation therapy at the local rehabilitation center, including speech therapy and traditional Chinese acupuncture. The patient demonstrated good compliance and attended follow-up examinations regularly. At the 3-month follow-up, lateral deviation of the tongue had largely disappeared, with a 70% recovery rate for hoarseness. At the 6-month follow-up, tongue protrusion was normal, and hoarseness recovery improved to 75%. At the 1-year follow-up, tongue protrusion remained normal, with hoarseness recovery reaching 80%. At the 2-year follow-up, the improvement rate for hoarseness further increased to 90%.

## Discussion

Tapia syndrome is a rare complication of endotracheal intubation with a low incidence rate. Once it occurs, it can severely impact a patient’s quality of life and may even be life-threatening in severe cases (3). Tapia syndrome refers to damage to the hypoglossal nerve (cranial nerve XII) and the recurrent laryngeal branch of the vagus nerve (cranial nerve X). It may occur during anterior or posterior cervical spine surgery, otolaryngological procedures, shoulder surgeries, and similar interventions (4–6). Symptoms include hoarseness, vocal cord paralysis, dysarthria, dysphagia, and tongue protrusion weakness/deviation. Spinal surgeries are predominantly performed under general anesthesia with endotracheal intubation, theoretically placing them at higher risk for Tapia syndrome (due to the large number of procedures). However, in practice, the incidence of Tapia syndrome is low and extremely rare. A literature review of medical databases including CNKI and PubMed (Table 1) revealed that Tapia syndrome associated with spinal surgery predominantly occurs in anterior and posterior cervical spine procedures, documented primarily as case reports. To date, no reports exist of Tapia syndrome arising from posterior lumbar spine surgery.

**Table 1:** Literature review of Tapia syndrome at home and abroad.

Author	Reported time	Sex	Age	Left/Right	Surgery	Onset time	Symptoms	Treatment measures	Follow-up	Recovery
Silva et al. (2)	2017	F	41	L	Right C5–6 and left C6–7 posterior foraminotomies	Day 1	Tongue deviates to the left, hoarseness, difficulty swallowing	Speech therapy, vocal cord medialization injections	1 year	Partial
Kerolus et al. (7)	2018	F	48	L	C2–3 ACDF	Day 7	Tongue deviates to the left, difficulty swallowing, difficulty speaking	Swallowing rehab	6 weeks	Complete
Shi et al. (8)	2020	F	59	R	C3–7 laminoplasties	Day 1	Tongue deviates to the right, articulation disorder	Corticosteroids, neurotrophic drugs	10 months	Complete
Waits et al. (9)	2020	M	52	L	C3–7 laminoplasties	Day 1	Voice paralysis, tongue deviation to the left, difficulty swallowing	Corticosteroids	3 months	Complete
Yan et al. (10)	2026	M	73	R	C4–6 ACCF	Day 1	Hoarseness	Corticosteroids, rehabilitation	Day 40	Almost complete

The etiology of Tapia syndrome is multifactorial. Most reported cases mention prior general anesthesia and endotracheal intubation procedures. Some scholars believe the primary causes are endotracheal intubation, improper techniques during the procedure, and incorrect head positioning post-intubation (11). The relatively narrow space between the soft tissues of the pharynx and the vertebral bodies may predispose patients to Tapia syndrome during invasive procedures (9). This risk is particularly heightened during posterior cervical surgery, where neck flexion further compresses this already confined space, increasing pressure on local structures (6, 12). Some researchers report (6) that maintaining a distance of ≥7 mm between the mandible and the anterior vertebral body may prevent Tapia syndrome, though this requires preoperative fluoroscopy. This patient developed hoarseness and lateral deviation of the tongue on the first postoperative day following lumbar PLIF surgery, diagnosed as Tapia syndrome. After aggressive treatment and 2 years of follow-up, the patient recovered to approximately 90% of preoperative status, though residual hoarseness persists. Based on this case, potential causes of Tapia syndrome include: ① The patient had a medium build with a relatively short, thick neck; preoperative cervical fluoroscopy was not performed, suggesting possible underlying pathology; ② Improper intubation or tube displacement during general anesthesia compressing the adjacent vagus and hypoglossal nerves; ③ Improper head/neck positioning during prone surgery; ④ Prolonged anesthesia and surgical duration leading to excessive compression.

Spinal surgery typically employs the prone position, with patients generally under general anesthesia with endotracheal intubation. During the procedure, Tapia syndrome may occur due to factors such as patient positioning or intubation. To effectively prevent Tapia syndrome, a series of preventive measures can be implemented: ① Perform intubation and related procedures gently, avoiding forceful maneuvers; ②Secure the tube properly and monitor the position of the endotracheal tube, adjusting it promptly if any abnormalities are detected; ③ Ensure proper head and neck positioning to prevent tube loosening from shifting; ④ Minimize anesthesia duration and shorten surgical time. Clear and effective execution at each stage, avoiding repetitive ineffective maneuvers, and heightened vigilance are essential to reduce or prevent Tapia syndrome.

Due to the relative rarity of Tapia syndrome in clinical practice, it must be differentiated from conditions such as Wallenberg syndrome, stroke, brainstem lesions, and intracranial tumors. Wallenberg syndrome (posterior lateral medullary syndrome) primarily manifests as vertigo, nausea/vomiting, nystagmus, dysphagia, dysarthria, ipsilateral Horner’s syndrome, and crossed sensory deficits. Its pathophysiology stems from occlusion of the posterior inferior cerebellar artery or vertebral artery, with cranial MRI aiding differential diagnosis. Stroke patients often have underlying conditions such as hypertension and hyperlipidemia, with sudden onset. Beyond hoarseness and tongue deviation, they frequently exhibit neurological localization signs like hemiplegia, sensory deficits, and dysarthria. Cranial CT or MRI scans can identify the responsible lesion. Brainstem lesions such as infarction, hemorrhage, or inflammation may also involve the hypoglossal and vagus nerves, causing similar clinical symptoms. However, patients typically exhibit concomitant damage to other cranial nerves. Patients with intracranial tumors typically have a prolonged course, often presenting with headaches, vomiting, papilledema, or other signs of increased intracranial pressure, alongside neurological deficits. Imaging studies reveal space-occupying lesions. Additionally, differential diagnoses include recurrent laryngeal nerve palsy and isolated hypoglossal nerve injury: recurrent laryngeal nerve palsy primarily manifests as hoarseness and abnormal vocal cord movement, generally without tongue deviation; isolated hypoglossal nerve injury presents mainly with tongue deviation and tongue muscle atrophy, without hoarseness or vocal cord paralysis indicative of vagus nerve involvement. A definitive differential diagnosis is typically achievable through detailed medical history, thorough physical examination, and necessary imaging and neurophysiological studies. This patient developed symptoms postoperatively, presenting primarily with hoarseness and tongue deviation, without other neurological localization signs such as limb movement disorders or sensory abnormalities. Cranial CT/MRI revealed no new cerebral infarction or space-occupying lesions, thus ruling out these conditions. Considering the history of general anesthesia with endotracheal intubation and prone position surgery, the diagnosis of Tapia syndrome is established.

Once diagnosed with Tapia syndrome, early and effective intervention is crucial for promoting rapid recovery. Of course, literature reports (2) indicate that the prognosis of Tapia syndrome depends on the extent of nerve damage, with 30% of patients achieving complete recovery, 39% experiencing partial recovery, and the remaining patients potentially exhibiting persistent symptoms. Treatment for Tapia syndrome (8) includes corticosteroids, fluid removal to reduce swelling, and neurotrophic drugs. Acupuncture, as an effective and safe treatment option (13), is also applicable for Tapia syndrome patients. Additionally, patients should initiate specialized rehabilitation training—such as articulation therapy and dysphagia therapy—under the guidance of a rehabilitation physician as early as possible. Active and effective intervention is crucial for patient recovery, reflecting the application of the accelerated recovery concept in spinal surgery (14).

## Conclusion

Clinically, Tapia syndrome has a low incidence rate, yet it significantly impacts patients’ quality of life when it occurs. Notably, there are currently no reported cases of Tapia syndrome developing after lumbar spine surgery, making it more prone to being overlooked. Therefore, enhancing awareness and understanding of Tapia syndrome is crucial. This will help prevent misdiagnosis and missed diagnosis, enable early intervention and treatment, thereby improving perioperative safety and achieving the goal of accelerated recovery.

## Data Availability

The original contributions presented in the study are included in the article/supplementary material, further inquiries can be directed to the corresponding author.
